# Progress in Obstetrics

**Published:** 1900-12-01

**Authors:** 


### Progress in Obstetrics.

**Placenta Prævia.**—Dr. Ford Anderson,[1] in discussing the treatment of this dangerous complication, says that although in some cases it has been advised to temporise until the child is viable, this is only justifiable if the hæmorrhages are neither frequent nor great, and where the patient can be kept under constant skilled supervision in a hospital; but considering the small chance to the child, and the great risk to the mother, from waiting, it is better, when once the diagnosis has been made, to empty the uterus as quickly as is possible compatable with safety. He strongly advocates the use of Champetier de Ribes' bag. This bag is in the shape of an inverted cone, the apex being at the os internum: it imitates the bag of membranes, forming a uniform and gentle dilator of the os uteri, the base of the larger bag being of such a size that when it has passed through the os uteri delivery can be completed. By traction on the pipe through which it is filled the uterine sinuses can be compressed, and, if necessary, the traction can be maintained for many hours by a light weight. Before introduction the bag should be scrubbed with soap and water and then soaked in carbolic solution 1–20. This, Dr. Anderson considers, is the rival treatment to sponge tents, and even to turning. Referring to the objections which have been raised to the use of the bag, he remarks that, in order to avoid septis, it is well to begin by removing clots from the cervix by an antiseptic douche. Although it has been said that the bag may cause rupture of the uterus, no case has been recorded. It is well, however, to ascertain the capacity of the bag, and to fill it slowly, stopping during uterine contraction. There might be some risk of this accident in the induction of premature labour, but here a small-sized bag might be used in the first instance. There is no doubt that displacement of the presenting part might be caused by the bag, but this could be rectified on withdrawal if not before. Already statistics show an improvement as regards infantile mortality, with no increased risk to the mother.

**Rupture of the Uterus.**—Rupture of the uterus is justly regarded as one of the most formidable and fatal accidents to which the pregnant woman is liable: it is a surgical complication of the first magnitude the outcome of which depends in no small measure upon the judgment and technical knowledge commanded by him who attempts to relieve the woman. A. W. Wendel,[2] in a very instructive paper on this subject, says that rupture of the uterus is far more frequent than is generally supposed, cases occurring in private practice if fatal from hæmorrhage being reported as post-partum hæmorrhage, and others merely as septic peritonitis when death results from infection. After discussing the causes and symptoms of this accident he sums up the treatment under the following headings:—(1) In threatened uterine rupture the woman is to be delivered at once by the method which does not increase the excessive intrauterine tension. (2) After rupture has occurred delivery by the natural passages is permissible only if the uterine injury is not aggravated thereby. (3) If the true conjugate diameter of the pelvic inlet measures less than 8 cm. a completely intraperitoneal fœtus should be delivered by abdominal section. (4) But if the conjugate measures 8 cm. or more, the uterus should be removed by vaginal hysterectomy, and the child delivered by the feet through the natural passages. (5) If life is detected in the child after the mother's death, post-mortem abdominal section should be performed. (6) The uterine tamponade is useful only as a dressing to prevent intestinal prolapse, or to retain reposited loops of intestine within the abdomen until the case can be submitted to operation. (7) Conservative surgery exposes the woman to the serious risk of subsequent rupture, and should therefore not be elected unless expressly commanded by the patient or immediate relatives. (8) Vaginal suture is open to the objection that bleeding vessels in the parametrium are apt to be overlooked. (9) When laparotomy becomes necessary for the birth of the fœtus, the uterus should be removed by supravaginal amputation, with extra- or intra-peritoneal treatment of the stump. (10) Vaginal hysterectomy is the elective procedure in all cases, but should be supplemented by abdominal section whenever necessary. Weiss and Schuhl [3] record two cases of complete rupture of the uterus during labour, in which supravaginal hysterectomy was performed, with fixation of the stump in the wound. They believe that even when the rupture is of limited extent, it is best to open the abdominal cavity and suture the rent from the peritoneal aspect, as this method allows of the possible discovery of extensive laceration of the peritoneum, and other damage which may otherwise be overlooked. Dr. R. D. Purefoy[4] reports two cases of rupture of the uterus in which the uterine cavity was packed with iodoform gauze: one case died, and the post mortem revealed a rent in the uterine wall allowing a finger to pass into the peritoneal cavity. There were three pints of blood-stained fluid in the peritoneum, and some lymphy deposit on the distended intestines. In the other case the gauze was removed on the third day, and a steady convalescence followed.

**The Treatment of Puerperal Septicæmia.**—There is still considerable difference of opinion as to the treatment of puerperal fever, especially as regards two of the more recent methods advocated, viz., the use of antistreptococcic serum and hysterectomy. With reference to the former, most varying opinions have been expressed. Louis A. Hering[5] believes that although the streptococcus is the chief source of mishief, other organisms such as the staphylococcus and the bacillus coli communis are often present: he maintains that the bacillus typhosus which some have found in puerperal infection is always the bacillus coli. He advocates the use of antistreptococcic serum in those cases in which it is proved that the infection is due to this organism, and believes that failures in the action of the serum may be attributed to the following causes: (1) Old serums; (2) strictly antistreptococcic serum can only be efficient against toxines produced by streptococci: it must be useless where we have a mixed infection to deal with; (3) delay in inaugurating treatment with the serum and administering it in insufficient quantities; (4) over-stimulation of the patient. All his cases were curetted, and the uterine cavity irrigated with a weak bichloride of mercury solution followed by a daily intrauterine hot douche of the same solution. He condemns the use of morphia, because it tends to interfere with the excretion of the toxines. The administration of the serum should be commenced at as early a period as possible, and a stimulant given immediately before its use. Alex. J. Anderson[6] reports a case of puerperal septicæmia in which the serum was used with success, but had a markedly depressant effect upon the circulation. Five injections were given, and after each the pulse became slow, irregular, and intermittent; and he insists that the effects of the serum should be watched from hour to hour night and day, on account of this depressing effect on the circulation, which he says lasts for several days after all injections have been stopped. Macharg[8] reports the result of his investigations in fifty cases of puerperal septic infection. Thirty of these died, and in twenty-one a post-mortem examination was obtained. Endometritis was the lesion most prominently found in these patients. Peritonitis was present in nine cases. In eight cases pus was found. Septic thrombosis of the uterine sinuses was present in six cases. As regards treatment, antiseptic intrauterine douches were given twice daily, and the uterus was well packed with iodoform gauze. Serum treatment was negative in its results. Harcourt Gervis[7] reports a case of puerperal septicæmia successfully treated by the antistreptococcic serum, and he maintains that no one should be satisfied when treating a case of puerperal fever unless in addition to the ordinary methods of treatment he employs the antistreptococcic serum, beginning, of course, at the earliest possible date. In this and every other case, however, the uterine douche, curetting, &c., have been used in addition to the serum, and it is difficult to say to what extent the recovery may be due to these measures. With regard to the other recent method of treatment, viz., hysterectomy, there is still greater difference of opinion; most writers, however, condemn the practice. Vinebery[9] has written a long paper on the subject, in which he emphasises the following points: (1) Puerperal sepsis is wound fever or wound infection, and this in the female genital canal, as elsewhere, calls for surgical measures, such as free drainage, irrigation, and the removal of any *débris* or exudate that may form on the surface of the wound. These measures failing to accomplish the desired result, ablation of the diseased organ is indicated as a *dernier ressort.* (2) In a given case of puerperal sepsis a thorough search is to be made of the whole of the genital canal in order to determine the site of the original infection. (3) If it is situated in the uterus, curettage, drainage, and irrigation are to be employed. In 95 per cent. of the cases of puerperal sepsis nowadays met with this plan of procedure will be all that is necessary to bring about a cure. (4) In the remaining 5 per cent., roughly speaking, these measures will not be efficacious in arresting the progress of the infection. An exploratory laparotomy is then indicated, the further course to be guided by the pathological lesions found. In most of these cases total hysterectomy will, he thinks, be required. Prochownick[10] contributes a very interesting paper on this subject. He has found treat ment by the serum useless, and has directed his attention to the problem of finding when the septic puerperul uterus should be extirpated. He sums up his experience by stating that he cannot decide upon extirpation of the uterus from the result of examination of matter taken from the uterus or vagina, but considers that examination of the blood is of far more importance. If general pyæmia be present, extirpation of the uterus is not indicated; if, however, it be evident that the uterus alone is pyæmic, then its removal may be followed by a good result.

**Brow Presentations.** — Schatz[11] believes that an essential element in causing brow presentation is spasm, or stricture of the lower uterine segment. The exact cause of this is difficult to find, as it does not seem to depend upon any condition in the pelvis, nor upon the shape of the child's head. Edgar[12] considers these cases the most difficult and dangerous of the various presentations of the head. The chief difficulty in the mechanism of labour is that the greatest diameter of the head is thrown across the brim of the pelvis. If conversion into a face or vertex presentation do not occur, either spontaneously or artificially great moulding of the head is necessary before the brow can become engaged, and in the majority of cases further progress is impossible. It is important, therefore, to understand the treatment of such cases, and Edgar describes the methods he recommends under the following headings: Firstly, when the head is movable above the brim a trial might be made of the postural method, although he prefers to adopt Schatz's, and failing this, Thorn's method; if both of these are unsuccessful he would recommend podalic version. Secondly, when the head is engaged in the brim, he would anæsthetise the patient and endeavour by Thorn's or Bandelocque's methods to convert the brow into a face or vertex presentation; if these, however, were unsuccessful version or the forceps might be cautiously tried. In intractable cases craniotomy would be required. Thirdly, when the brow has descended to or nearly to the pelvic floor Bandelocque's method of flexing the head should be tried, and failing that the chin ought to be brought downwards and forwards. If, however, the child be dead, or the mother's condition demand it, craniotomy should be resorted to.

**Post-partum Hæmorrhage.**—E. P. Davis,[13] writing on this subject, says that in spite of recent advances in modern obstetric science, post-partum hæmorrhage remains one of the great dangers to the parturient woman. The most common cause of post-partum hæmorrhage is inefficient contraction of the uterus from exhaustion, less frequent causes being retention of a partially detached placenta, or of a portion of placenta or membranes within the uterus, lacerations of the genital tract, and a profoundly altered condition of the patient's blood. The prevention of post-partum hæmorrhage calls for the careful avoidance of exhaustion. During the first stage of labour the patient should receive liquid food in small quantities as frequently as she can take it. In the second stage as soon as the patient begins to show signs of exhaustion, interference is indicated. If an anæsthetic is necessary, Dr. Davis thinks ether preferable to chloroform. The uterus should be carefully followed down as the child is delivered, and if it fails to contract promptly and vigorously, a hot douche of sterilised water or saline solution (temperature 110–115° F.) should be given, and at the same time the patient should have a hypodermic injection of ergot and strychnine. The head should be kept low, and if the pulse is lacking in volume a rectal injection of freshly-made coffee well diluted will be of advantage. Dr. Davis lays great stress on the importance of the prevention of post-partum hæmorrhage, believing that, like septic infection, its prevention is more satisfactory than the actual treatment of the condition. In the event of active hæmorrhage occurring the physician must quickly differentiate between a relaxed condition of the uterus and hæmorrhage from a tear in the cervix, pelvic floor, or vaginal tissues. If the hæmorrhage is due to a relaxed condition of the uterus, that organ should be grasped by the hand, and a douche of hot sterile water (temperature 110° F.) immediately given; should this fail to make the uterus contract, Dr. Davis recommends that it should be at once packed with aseptic or iodoform gauze. If the patient is kept externally clean by antiseptic solutions, and if sterile napkins are worn, the gauze packing may safely remain undisturbed for twenty-four hours. It should then be removed and the uterus thoroughly, but very gently, douched with a saline solution at a temperature of 110° F.

[1] Lancet, June 9, 1900. [2] Med. Rec., May 26, 1900. [3] Brit. Med. Jour., June 16, 1900. [4] Dublin Jour. Med. Sci., May, 1900. [5] New York Med, Jour., April 7, 1900, [6] Brit. Med. Jour., Feb. 17, 1900. [7] Brit. Med. Jour., May 26, 1900. [8] Brit. Med. Jour., Feb. 17, 1900. [9] Amer. Jour. Med. Sci., Feb., 1900. [10] Amer. Jour. Med. Sci., Feb., 1900. [11] Amer. Jour. Med. Sci., May, 1900. [12] Glas. Med. Jour., March, 1900. [13] Med. Rec., June 13, 1900.

